# A suitable enzymatic method for starch quantification in different organic matrices

**DOI:** 10.1016/j.mex.2019.09.040

**Published:** 2019-10-04

**Authors:** Breno de Castro Silva, Letícia Artuzo Godoi, Sebastião de Campos Valadares Filho, Diego Zanetti, Pedro Del Bianco Benedeti, Edenio Detmann

**Affiliations:** Department of Animal Science, Universidade Federal de Viçosa, Viçosa, Minas Gerais, 36570-900, Brazil

**Keywords:** An enzymatic method for starch analysis, Feed analysis, Glucose, Ruminant faeces, Ruminant digesta

## Abstract

Starch can represent 70–80% of the cereals grains (on a dry matter basis) used for livestock feeding. Several methods have been developed to estimate the feed starch contents of energy feed sources. However, the efficiency of these methods to evaluate the starch content in other feed sources, as well as other types of samples used to evaluate starch availability in the gastrointestinal tract, such as digesta and faeces, remains unclear. Furthermore, most of the currently used starch analysis methods have not been effectively evaluated, being only applied to samples of sporadic experiments, without a wide-ranging validation of the procedures and results. Here, we propose a modification of a method for analysing the starch content in different organic matrices normally evaluated in ruminant nutrition studies. The evaluated organic matrices were: soybean meal, soybean hull, Tifton 85 Bermuda grass hay, abomasal digesta, and faeces.

• The modified method is more feasible than the original procedures.
• The modified method estimates the starch contents in different organic matrices with accuracy and precision.

## Graphical abstract

**Figure:**
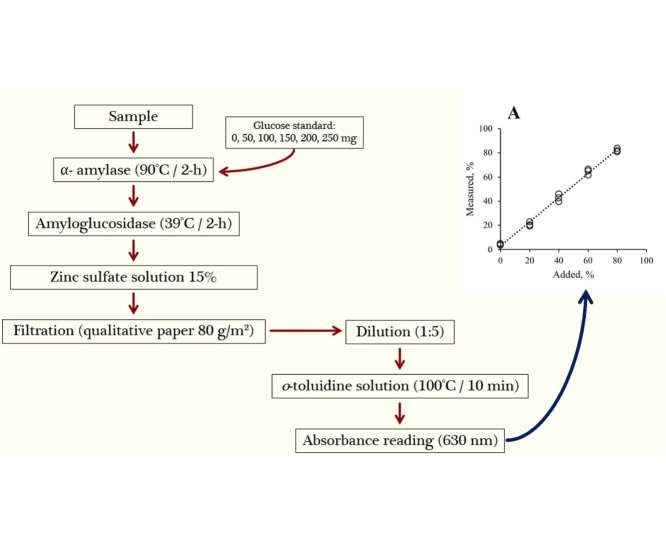

**Specification Table**

**Table:** 

Subject Area:	Agricultural and Biological Sciences
More specific subject area:	Feed analysis applied to animal science
Method name:	An enzymatic method for starch analysis
Name and reference of original method:	R. Zinn, Influence of flake density on the comparative feeding value of steam-flaked corn for feedlot cattle, Journal of Animal Science 68 (1990) 767-775.
Resource availability:	If applicable, include links to resources necessary to reproduce the method (e.g. data, software, hardware, reagent)

## Method details

### Location and ethical approval

The experiment was carried out in the Department of Animal Science at the Universidade Federal de Viçosa, Viçosa, Minas Gerais, Brazil. The care and handling of the experimental animals followed protocols that were approved by the Institutional Animal Care and Use Committee of the Universidade Federal de Viçosa (protocol number 32/2018).

### Original method

This study proposes several modifications to the starch analysis method proposed by Zinn [1], which has the following steps:

1 Weigh 200 mg (air-dried basis) of sample, previously grounded to pass through a 2-mm screen sieve in a knife mill, into a 20-mL screw-cap test tube;
2 Add 10 mL of distilled water and 10 mL of the buffer solution (9.91 g/L of anhydrous sodium acetate and 7.27 mL/L of glacial acetic acid);
3 Add 67 units of amyloglucosidase (1 mg of enzyme) and 1 drop of toluene;
4 Tightly cap the tube, gently shake it and incubate at 39 °C for 2 h in shaking water bath;
5 Transfer 1 mL of the starch hydrolisate solution and 4 mL of the trichloroacetic acid solution (TCA; 30 g/L) into a 10-mL centrifuge tube, then vortex briefly;
6 Keep tube at room temperature for 5 min and then centrifuge at 6000 rpm for 10 min;
7 Add 4 mL of *o*-toluidine solution (60 g/L o-toluidine solution in glacial acetic acid) and 400 μL of TCA to the supernatant solution in a separate test tube. Then, cap and incubate it at 100 °C in a water bath for 10 min;
8 Remove the tube from the water bath and place it in an ice bath for 5 min;
9 Read absorbance at 630 nm.

Compromising points regarding these analysis procedures and method accuracy were properly studied and will be further discussed.

### Organic matrices and statistical analysis

A starch recovery test was performed using five different matrices: soybean meal, soybean hulls, Tifton 85 hay, and cattle abomasal digesta and faeces. Samples of abomasal digesta and faeces were collected from one Nellore bull (330 kg of body weight) fed a Tifton 85 grass hay-based diet, and oven dried (55 °C). All materials were ground to pass through a 1-mm screen sieve (Wiley mill; Thomson Scientific Inc., Philadelphia, PA). Five levels of soluble starch (101252, Merck, Darmstadt, Germany) were added over each matrix: 0, 20, 40, 60, and 80% (as-is basis). For the calculations, the amount of starch added was corrected according to its moisture content (10%). Starch analyses were performed in triplicate for each matrix/starch level.

Results for each matrix were evaluated using a simple linear regression model of the measured (dependent variable) over actual (independent variable) starch added, according to the following model:

(1) $${\text{Y}}_{\text{ij}}={\text{β}}_{0}+{\text{β}}_{1}\times {\text{X}}_{\text{i}}+{\text{e}}_{(\text{i})\text{j}},$$

where Y_ij_ is the observed starch content of the i^th^ level of starch inclusion in the j^th^ replicate; β_0_ is the intercept, which represents the basal content of starch in the matrix; β_1_ is the slope, which corresponds to the recovery of added starch; X_i_ is the level of starch inclusion; and e_(i)j_ is the random error assumed to have a normal distribution [e_(i)j_ ∼ N (0, σ^2^)].

Model (1) was evaluated using the following hypotheses:

(2) $${\text{H}}_{0}:{\text{β}}_{1}=\text{1 vs}\text{.}{\text{H}}_{\text{a}}:{\text{β}}_{1}\neq 1$$

The acceptance of the null hypothesis described in Eq. (2) implies a total recovery of the starch added over the matrix. Besides this first statistical evaluation, a test for linear model identity was applied to the total data set to identify differences regarding starch recovery among the evaluated matrices using a regression with "dummy" variables [2]. A likelihood ratio test [3] was used to verify whether the starch recovery was complete and similar across all the evaluated organic matrices. All analyses were performed using the REG procedure of SAS 9.4 (Statistical Analysis System Institute, Inc., Cary, NC, USA) and significance was established at *P* <  0.05.

### Comparison between methods

After the evaluation of modified method on the different organic matrices, a comparative evaluation was performed where six samples were analysed following the original Zinn [1] method and the new approach proposed here. Samples of abomasal digesta and faeces were taken from two animals fed different diets: a diet containing 70% of concentrate feeds (maize and soybean meal) and 30% of forage (in a dry matter basis), and a whole grain diet (most maize grain, without forage). Samples of maize and sorghum grains were also evaluated due their importance as starch sources for livestock production. Four replicates of each sample were evaluated by both methods.

### Modified method evaluation

Initially, in our laboratory, several samples of feeds, faeces, and digesta were analysed using the original method [1]. However, the results were compromised (data not shown) due to the lack of a proper description of the methodology, along with other constraints that will be properly discussed throughout this section. Thus, adaptations were necessary to ensure an improved performance of the procedures and the adequacy of the results in terms of sensibility, precision, and accuracy. A summary, including all modified method procedures, is described in the Appendix section.

First, the sample amount was increased to 250 mg (air-dried basis) to improve the absorbance reading. In the evaluations routinely performed in our laboratory, 200-mg samples were not enough to assure an adequate absorbance with a reliable signal-to-noise ratio. Additionally, all samples were ground to pass through a 1-mm screen sieve to increase specific surface area for more effective enzymatic action, which is expected to improve the precision of results.

Steps 2–4 were restructured to optimise the amyloglucosidase action. First, 10 mL of distilled water was added into the test tubes, as described in the original method. Nonetheless, 0.5 mL of a thermostable α-amylase solution (Lyquozime Supra 2.2X, Novozymes; Araucária, PR, Brazil) was also added into the tubes. Then, the tubes were incubated at 90 °C for 2 h in a water bath and subsequently ice bathed for 10 min. The inclusion of this step aimed to establish a partial process of starch hydrolysis, which prevents its gelatinisation [4] and optimises amyloglucosidase subsequent action, which only acts slowly on native starch [5]. As exposure time may affect amyloglucosidase activity, sample pre-exposure to thermostable α-amylase might increase further starch recovery, especially in high-starch samples. Thereafter, 10 mL of the buffer solution (original method step 2) and 0.5 mL of amyloglucosidase solution (AMG 300 L, Novozymes; Araucária, PR, Brazil) were added to the tubes. The AMG 300 L, an industrial enzyme with standardised activity [6], was chosen due to the lack of information about this enzyme in the original method [1]. Then, tubes were incubated at 39 °C for 2 h in a water bath and subsequently ice bathed for 10 min. For water bath incubations, non-shaking equipment was used to increase feasibility. However, tubes were manual shaken every 30 min.

Regarding step 5 of the original method, the 4 mL of TCA solution used for protein precipitation was replaced with 2 mL of 15% zinc sulphate solution [7], which is more stable at room temperature and easily stored. The centrifugation procedure from step 6 was replaced by solution filtration through qualitative filter paper (80 g/m²) to increase method accessibility.

The *o*-toluidine solution (*o*-toluidine 0.6 M in glacial acetic acid, containing thiourea as a stabiliser; Sigma T1199, Sigma Chemical Co., St. Louis, MO) was used for glucose quantification, since the original method does not specify a reagent for this procedure [1]. The *o*-toluidine solution (4 mL) was added to 400 μL of filtrate, according to the original method [1]. However, the solution had an extremely dark green colouration, compromising the analysis sensitivity (i.e. absorbance). To solve this problem, filtrate was diluted with distillate water (1:5 mL) for all samples, including blanks and standards. Then, 4 mL of the *o*-toluidine solution was added to this diluted solution.

**Fig. 1:**
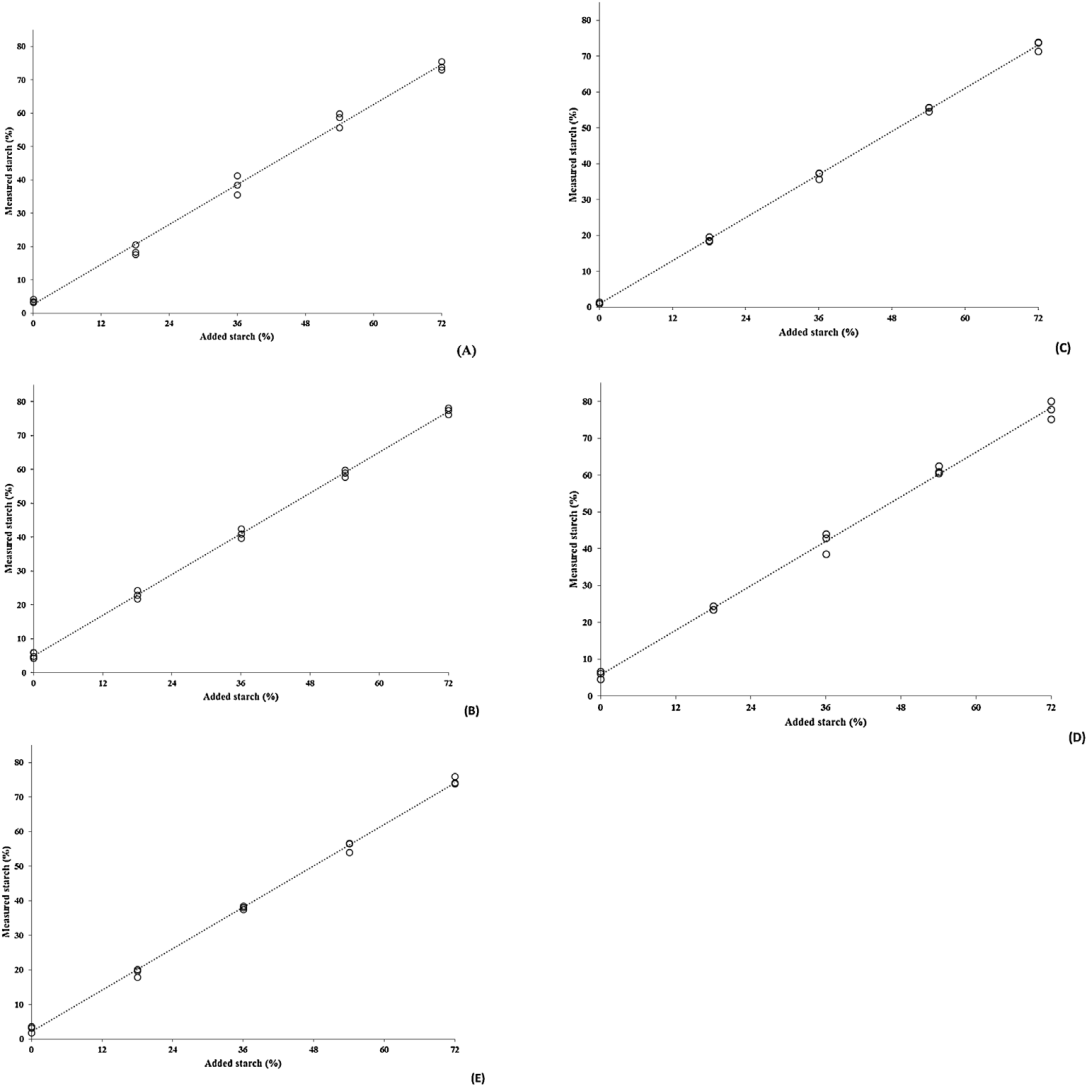
Relationship among starch added and measured starch contents (For details about the relationships, please see Table 1, A = soybean hulls, B = soybean meal, C = Tifton 85 hay, D = abomasal digesta, E = faeces).

**Fig. 2:**
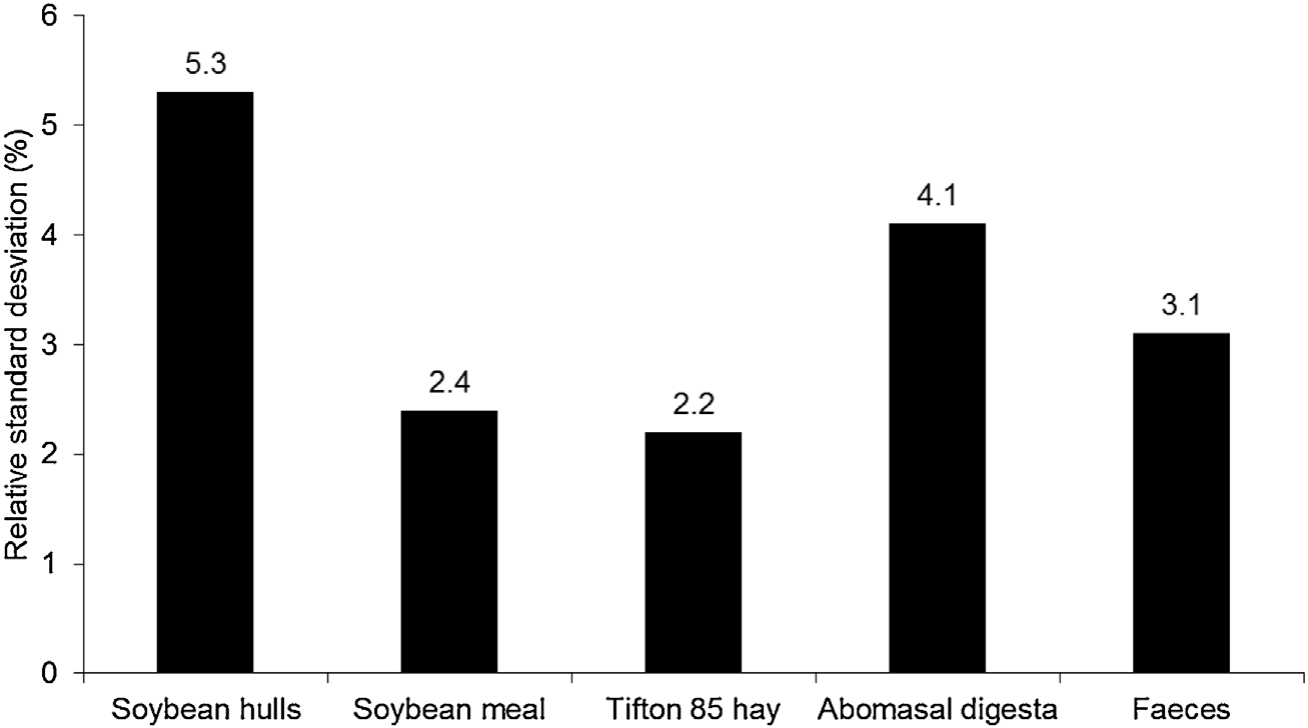
Relative standard deviation for starch contents analysed in different organic matrices (Relative standard deviation = residual standard deviation/average starch concentration).

All organic matrices had a complete recovery of added starch when the modified method was performed (*P* ≥  0.67, Table 1, Fig. 1A–E). Furthermore, the likelihood ratio test has shown similarity regarding starch recovery among matrices (*P* >  0.99). Thus, the modified method estimated the starch added to the evaluated organic matrices with accuracy. Similarly, the relative standard deviations for the starch content had a small range (from 2.2–5.3%; Fig. 2), which indicates that the modified method estimated the starch contents precisely.

**Table 1:** Simple linear regression parameters estimated from measured (dependent variable) over actual (independent variable) starch added on different organic matrices.

Matrix	Regression parameters				
	Intercept (β_0_)	Slope (β_1_)	S_xy_	r^2^	*P*-Value^a^
Soybean hulls	2.601 ± 0.916	1.001 ± 0.021	2.05	0.994	0.954
Soybean meal	4.995 ± 0.445	1.001 ± 0.010	0.99	0.999	0.917
Tifton 85 hay	0.955 ± 0.363	1.001 ± 0.008	0.81	0.999	0.852
Abomasal digesta	5.784 ± 0.775	1.008 ± 0.017	1.73	0.996	0.675
Feces	2.113 ± 0.521	1.000 ± 0.012	1.16	0.998	0.991

a H_0_: β_1_ = 1 vs. H_a_: β_1_ ≠ 1. Slope coefficient is related to the recovery rate of added starch (β_1_ = 1 means complete recovery).

### Comparison between methods

**Fig. 3:**
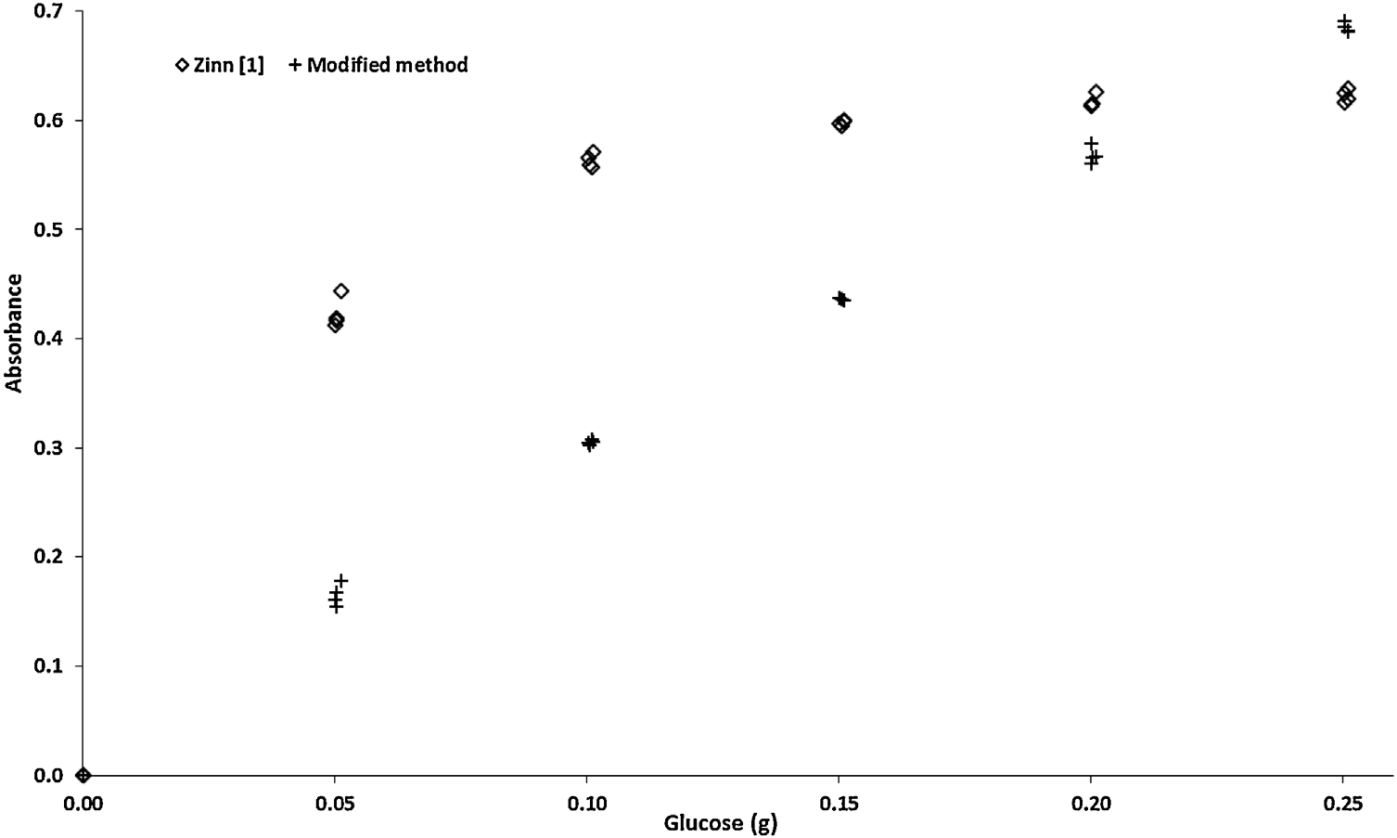
Relationship between amount of glucose and absorbance of the standard solutions obtained by using the original Zinn [1] method and the modified method.

The shapes of the glucose standard curves obtained by both methods were quite different to each other (Fig. 3). While the modified method produced a linear relationship between glucose amount and absorbance, which indicates an accordance with the Lambert-Beer Law [8], the original method described a curvilinear shape for that relationship. There are several reasons for observing deviations from the Lambert-Beer Law. However, in our specific case, the deviation by using Zinn [1] method seems to have chemical causes. As previously discussed, when applying Zinn [1] method we observed as extremely dark green in the solutions following the *o*-toluidine addition. Such a pattern should indicate a high concentration of the analyte in the solution, which was outlined by the additional dilution with distillate water. Actually, as analyte concentration increases, the intermolecular distances in a given sample solution will decrease, eventually reaching a point at which neighbouring molecules mutually affect the charge distribution of the other. This perturbation may significantly affect the ability of the analyte to capture photons of a given wavelength; that is, it may alter analyte absorptivity [9]. This will cause the linear relationship between concentration and absorbance to break down since absorptivity term is the constant of proportionality in Lambert-Beer Law [8,9]. This argument can explain why the behaviour of the Zinn [1] standard curve closed to a flat shape as glucose amount in the solution increases (Fig. 3). Overall, this pattern may reduce the sensibility of the method and compromise estimates, mainly for high-starch samples.

General, our arguments are supported by the estimates of starch contents in maize and sorghum grain samples (Table 2). The contents obtained by original Zinn [1] method were lower compared to the modified method. Actually, those estimates were unlikely considering the structure of the grains, whose estimates obtained by using the modified method were much more realistic when considering the starch content naturally expected. The same pattern was observed for abomasal digesta, where higher starch contents were obtained by using the modified method. On the other hand, an unexpected pattern was verified for faecal starch when Zinn [1] method was applied. The whole-grain diet presented less starch than 70% concentrate diet. The starch is a non-fibrous carbohydrate what is expected to present a high and relatively constant true digestibility [10]. Considering this, it should be established that amount of undigested starch in faeces be proportional to the amount of dietary starch [11]. Therefore, the whole-grain diet should have presented more faecal starch compared to 70% concentrate diet. However, when the modified method was applied, the expected pattern of faecal starch content was verified.

**Table 2:** Starch content estimates (% dry matter) obtained for different samples by using the original method of Zinn [1] and the modified method.

	Method^a^	
Sample^b^	Zinn [1]	Modified method
Maize grain	39.05 ± 1.42	75.09 ± 0.19
Sorghum grain	24.69 ± 0.67	71.64 ± 0.51
Abomasal digesta WG	41.14 ± 3.20	60.83 ± 0.50
Abomasal digesta 70	33.41 ± 1.02	16.30 ± 0.22
Faeces WG	24.08 ± 3.29	31.85 ± 1.18
Faeces 70	33.39 ± 1.26	17.16 ± 0.62

a Mean ± standard error.

b WG, samples obtained from an animal fed a whole-grain diet; 70, samples obtained from an animal fed a diet with 70% of concentrate (30% of forage in a dry matter basis).

For all evaluated samples the precision of the starch content estimates was higher for the modified method (Table 2). Such improvement in random variation seems to be a reflex of the several modifications we proposed along the original method.

In summary, the modified method has estimated the starch content in different organic matrices with accuracy, precision, and feasibility. Therefore, this modified method might be recommended for the evaluation of the starch contents of feeds and different materials obtained in digestion trials with ruminants, such as abomasum digesta and faeces.
