# Supplementary material for: A suitable enzymatic method for starch quantification in different organic matrices
Source: MethodsX. 2019 Oct 4;6:2322–8. doi: 10.1016/j.mex.2019.09.040 (PMC6812349; doi:10.1016/j.mex.2019.09.040)
Supplement: Supplementary file 1 [file mmc1.docx]

**Supplementary material *and/or* Additional information:**

# **Description of the modified method and calculation example**

#### *Reagents*

- Toluol (C_6_H_5_CH_3_)
- Zinc sulphate heptahydrate (ZnSO_4_ .7H_2_O)
- Sodium acetate anhydrous (C_2_H_3_NaO_2_)
- Glacial acetic acid (C_2_H_4_O_2_)
- D-glucose anhydrous
- α-amylase (Lyquozime supra 2.2x, Novozymes; Araucária, PR, Brazil)
- Amyloglucosidase (AMG 300L, Novozymes; Araucária, PR, Brazil)
- *o*-Toluidine solution 0.6 M in glacial acetic acid, containing thiourea as a stabiliser (Sigma T1199, Sigma Chemical Co., St. Louis, MO)

#### *Equipment*

- Analytical balance (0.0001g)
- Screw-cap test tubes (30 mL)
- Beaker (500 mL)
- Pipette (100 μL)
- Volumetric flask (1 L)
- Funnels
- Qualitative filter paper (80 g/m²)
- Test tubes (30 mL)
- Test tubes (10 mL)
- Water bath
- Ice bath
- Spectrophotometer

#### *Solution preparation*

- 15% zinc sulphate solution: Add 267.18 g of zinc sulphate heptahydrate into a 1 L volumetric flask, dissolve it in approximately 100 mL of distilled water, and fill up to the calibration mark with distilled water.
- Buffer solution: Add 9.91 g of sodium acetate anhydrous and 7.27 mL of glacial acetic acid into a 1 L volumetric flask, dissolve it in approximately 100 ml of distilled water and fill up to the calibration mark with distilled water.

#### *Procedures*

1. Weigh 0, 50, 100, 150, 200, and 250 mg of D-glucose anhydrous (standards) into 30-mL screw-cap test tubes. Standards must be running at the same time as the samples.
2. Weigh 250 mg (air-dried basis) of samples, previous grounded through a 1-mm screen in a knife mill, into 30-mL screw-cap test tubes;
3. Add 10 mL of distilled water, 0.5 mL of thermostable α-amylase, and 1 drop of toluene into each tube. Gently shake and incubate at 90°C for 2 h in a water bath (gently shake every 30 min). Place tubes into the water bath at room temperature and start counting time when the temperature reaches 90°C;
4. Remove tubes from the water bath and place them in an ice bath for 10 min;
5. Remove tubes from the ice bath. Then, add 9.5 mL of buffer solution and 0.5 mL of amyloglucosidase into each tube, gently shake and incubate at 39°C for 2 h in a water bath (gently shake every 30 min);
6. Remove tubes from the water bath and place them in an ice bath for 10 min;
7. Remove tubes from the ice bath and add 2 mL of 15% zinc sulphate solution into each tube. Then, vortex briefly and keep at room temperature for 10 min;
8. Using funnels, pass solution through qualitative filter paper into 30-mL test tubes;
9. Dilute 1 mL of the filtrate in 5 mL of distilled water, then hand shake tubes;
10. Pipette 100 μL of the diluted solution into 10-mL test tubes, add 4 mL of *o*-toluidine solution, cap tubes with marbles and incubate at 100 °C in a water bath for 10 min;
11. Remove tubes from the water bath and place them in an ice bath for 5 min;
12. Remove tubes from the ice bath and wait until tubes reach temperature stabilisation.
13. Read absorbance at 630 nm. Use the standard with 0 mg of glucose to set up the device, proceed with reading 50, 100, 150, 200, and 250 mg glucose standards, then read samples.

#### *Calculation example*

Sample weight (as-is): 0.2517g

Dry matter content: 89.32%

Reading solution absorbance: 0.300 nm

An example of absorbance for the glucose standards is presented in Supplementary Table 1.

**Supplementary Table 1.** Example of standards absorbance

| Item | Glucose, g | | | | | |
| --- | --- | --- | --- | --- | --- | --- |
|  | 0 | 0.050 | 0.100 | 0.150 | 0.200 | 0.250 |
| Absorbance, nm | 0.000 | 0.081 | 0.166 | 0.265 | 0.376 | 0.448 |

Steps:

1. Adjust the simple linear regression equation without an intercept according to the Lambert-Beer law (X = standards glucose amount; Y = absorbance):

$$\hat{\beta}=\frac{\sum_{i} (X_{i}\times Y_{i})}{X_{i}^{2}}=\frac{(0\times0.000)+...+(0.250\times0.448)}{0^{2}+...+0.250^{2}}=1.8007$$

1. Calculate the glucose released from starch in the sample:

$$\text{Glucose (g)}=\frac{\text{SA}}{\hat{\text{β}}}=\frac{0.300}{1.8007}=0.1667$$

Where SA is the sample absorbance.

1. Calculate the sample content of glucose released from starch:

$$\text{[Glucose]}_{\text{a}}=\frac{\text{Glugose(g)}}{\text{Sample(g)}}\times100=\frac{0.1667}{0.2517}\times100=66.19\%$$

$$\text{[Glucose]}_{\text{d}}=\frac{\text{[Glucose]}_{\text{a}}}{\text{DM}}\times100=\frac{66.19}{89.32}\times100=74.10\%$$

where [Glucose]_a_ is the glucose content in the sample as-is; [Glucose]_d_ is the glucose content in the sample on a dry matter basis, and DM is the dry matter content of the sample (%).

1. Convert the glucose into starch content (% of dry matter):

$$\text{[Starch]}=\text{[Glucose]}_{\text{d}}\times0.9=74.10\times0.9=66.69\%$$

The coefficient (0.9) is based on the fact that there is a loss of a water molecule when a α linkage is established between the glucose molecules to form the starch chain. On average, it implies a weight loss of 10%.

***References:**

1. R. Zinn, Influence of flake density on the comparative feeding value of steam-flaked corn for feedlot cattle, Journal of Animal Science 68 (1990) 767-775.

2. N.R. DrapeR, H. Smith, Applied regression analysis. New York, John Wiley & Sons, 1966.

3. C.R. Rao, Linear statistical inference and its applications. New York, John Wiley & Sons, 1973.

4. M.B. Hall, Methodological challenges in carbohydrate analyses. Revista Brasileira de Zootecnia 36 (2007) 359-367.

5. I.L. Batey, Starch analysis using thermostable alpha-amylases. Starch 34 (1982) 125-128.

6. D.I. Gomes, C.B. Sampaio, E. Detmann, S.C. Valadares Filho, R. Mezzomo R, J.G. Regadas Filho, Utilization of industrial enzymes in the evaluation of neutral detergent insoluble fiber content in high-starch samples. Semina: Ciências Agrárias 35 (2014) 2629-2642.

7. Xiong Y, Bartle S, Preston R. Improved enzymatic method to measure processing effects and starch availability in sorghum grain. Journal of Animal Science 68 (1990) 3861-3870.

8. D.A. Skoog, F.J. Holler, S.R. Crouch, Principles of instrumental analysis. 7^th^ ed. Boston: Cegage Learning, 2017.

9. M.H. Penner, Ultraviolet, visible, and flurescence spectroscopy. In: S.S. Nielsen, Food analysis. 2^nd^ ed. Maryland, Aspen Publishers, 1998. pp.397-412.

10. P.J. Van Soest, The nutritional ecology of the ruminant. Ithaca, Cornell University Press, 1994.

11. M. Caetano, R.S. Goulart, S.L. Silva, J.S. Drouillard, P.R. Leme, D.P.D. Lanna, Effect of flint corn processing method and roughage level on finnishing perfomance of Nellore-based cattle. Journal of Animal Science 93 (2015) 4023-4033.
